# Camelpox virus encodes a schlafen-like protein that affects orthopoxvirus virulence

**DOI:** 10.1099/vir.0.82748-0

**Published:** 2007-06

**Authors:** Caroline Gubser, Rory Goodbody, Andrea Ecker, Gareth Brady, Luke A. J. O'Neill, Nathalie Jacobs, Geoffrey L. Smith

**Affiliations:** 1 Department of Virology, Faculty of Medicine, Imperial College London, St Mary's Campus, Norfolk Place, London W2 1PG, UK; 2 School of Biochemistry and Immunology, Trinity College Dublin, Dublin 2, Ireland

## Abstract

Camelpox virus (CMLV) gene *176R* encodes a protein with sequence similarity to murine schlafen (m-slfn) proteins. *In vivo*, short and long members of the m-slfn family inhibited T-cell development, whereas *in vitro*, only short m-slfns caused arrest of fibroblast growth. CMLV 176 protein (v-slfn) is most closely related to short m-slfns; however, when expressed stably in mammalian cells, v-slfn did not inhibit cell growth. v-slfn is a predominantly cytoplasmic 57 kDa protein that is expressed throughout infection. Several other orthopoxviruses encode v-slfn proteins, but the *v-slfn* gene is fragmented in all sequenced variola virus and vaccinia virus (VACV) strains. Consistent with this, all 16 VACV strains tested do not express a v-slfn detected by polyclonal serum raised against the CMLV protein. In the absence of a small animal model to study CMLV pathogenesis, the contribution of CMLV v-slfn to orthopoxvirus virulence was studied via its expression in an attenuated strain of VACV. Recombinant viruses expressing wild-type v-slfn or v-slfn tagged at its C terminus with a haemagglutinin (HA) epitope were less virulent than control viruses. However, a virus expressing v-slfn tagged with the HA epitope at its N terminus had similar virulence to controls, implying that the N terminus has an important function. A greater recruitment of lymphocytes into infected lung tissue was observed in the presence of wild-type v-slfn but, interestingly, these cells were less activated. Thus, v-slfn is an orthopoxvirus virulence factor that affects the host immune response to infection.

## INTRODUCTION

*Camelpox virus* (CMLV) is a member of the genus *Orthopoxvirus* (OPV) of the family *Poxviridae*, a group of large double-stranded DNA viruses that replicate in the cytoplasm (Moss, 2001). Compared with other OPVs, CMLV is poorly characterized. The genomes of CMLV strains CMS (Gubser & Smith, 2002) and M-96 (Afonso *et al.*, 2002) were sequenced and revealed that CMLV is genetically closely related to variola virus (VARV), the cause of smallpox (Gubser & Smith, 2002). Like other OPVs, the CMLV genome has a highly conserved central region of about 100 kb encoding genes that are mostly essential for virus replication. In contrast, the genome termini encode proteins known or predicted to affect virus virulence or modulation of the host's immune response. Among these terminal genes, CMLV strain CMS *176R* encodes a protein with sequence similarity to mammalian schlafens (slfn) (Gubser & Smith, 2002).

**Fig. 1. f1:**
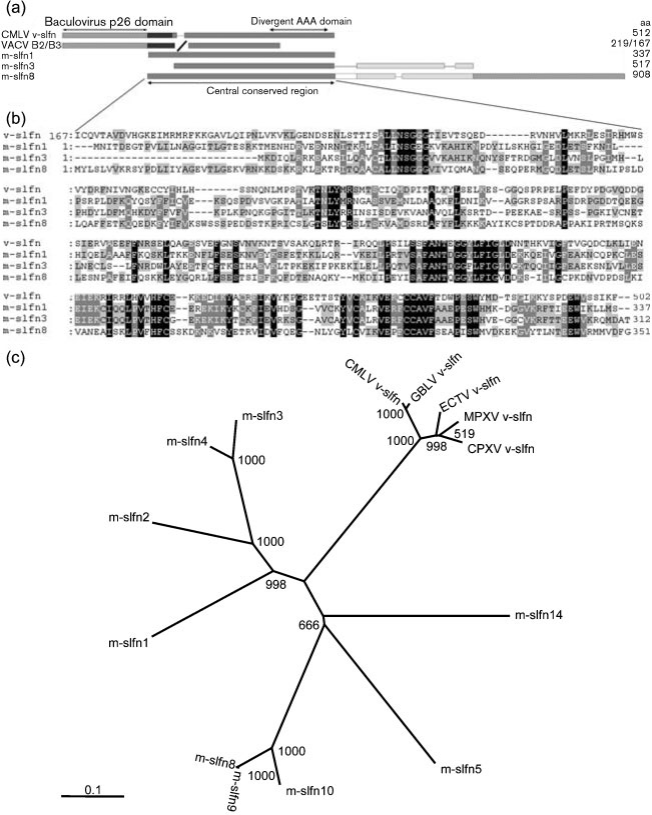
CMLV v-slfn is related to members of the mammalian slfn family. (a) Diagrammatic representation of CMLV strain CMS v-slfn (GenBank accession no. AAG37679), VACV-WR B2 (YP_233066) and B3 (YP_233067), and murine slfns m-slfn1 (AAH52869), m-slfn3 (NP_035539) and m-slfn8 (NP_853523). Protein sizes (number of aa) are shown on the right. (b) clustal w (Thompson *et al.*, 1994) alignment of the m-slfn1, 3 and 8 conserved region with the C terminus of v-slfn. Identical residues are shown in black and residues conserved in two or three out of four sequences are shown in light or dark grey, respectively. Amino acid co-ordinates are indicated. (c) Unrooted phylogenetic tree showing the relationship of v-slfn from camelpox virus (CMLV), cowpox virus (CPXV), monkeypox virus (MPXV), ectromelia virus (ECTV), taterapox virus (GBLV) and m-slfns. Protein sequences were aligned using clustal w and an unrooted tree was generated based on this alignment using phylip on the European Bioinformatic Institute website (http://www.ebi.ac.uk/clustalw/). Bootstrap values from 1000 replica samplings and the divergence scale (substitutions per site) are indicated.

The prototypic member of the slfn family, murine (m-)slfn1, was discovered by a subtractive hybridization between transgenic mice in which T-cell maturation was halted at the CD4^+^8^+^ double-positive stage and mice in which maturation was skewed toward CD4 single-positive selection (Schwarz *et al.*, 1998). blast searches identified a further eight related mouse genes that are classified as short (slfn1 and 2), intermediate (slfn3 and 4) or long (slfn5, 8, 9, 10 and 14) m-slfns, depending on their size (Schwarz *et al.*, 1998; Geserick *et al.*, 2004) (Fig. 1). All m-slfns share a conserved region that contains a putative divergent ATPase associated with the cellular activities (AAA) domain (Lupas & Martin, 2002; Frickey & Lupas, 2004), whereas intermediate and long m-slfns have additional C-terminal sequences. *In vivo*, short and long m-slfns inhibit T-cell development (Schwarz *et al.*, 1998; Geserick *et al.*, 2004), whereas *in vitro*, only m-slfn1 caused arrest of fibroblast growth by inhibition of cyclin D1 (Schwarz *et al.*, 1998; Geserick *et al.*, 2004; Brady *et al.*, 2005). Additionally, the expression level of m-slfns is regulated differentially after infection with the intracellular pathogens *Brucella* (Eskra *et al.*, 2003) and *Listeria* (Geserick *et al.*, 2004), suggesting a role in host defence against pathogens.

This study describes the characterization of CMLV strain CMS slfn-like protein 176 (v-slfn). Bioinformatic analyses showed that v-slfn is most closely related to short m-slfns. However, unlike m-slfn1, v-slfn did not inhibit cell growth when expressed stably in mammalian cells. Using specific antiserum, we showed that v-slfn is a predominantly cytoplasmic 57 kDa protein that is expressed throughout infection. Screening of 18 OPVs showed that immunologically related proteins of similar sizes are expressed by two cowpox virus (CPXV) strains but not by any of the 16 vaccinia virus (VACV) strains tested. To address whether v-slfn affected OPV virulence, CMLV v-slfn was expressed from the thymidine kinase (TK) locus of an attenuated VACV strain. *In vivo*, this recombinant virus was attenuated further compared with controls, and induced different recruitment of lymphocytes to the site of infection.

## METHODS

### Cells and viruses.

BS-C-1, TK^−^143 and HeLa cells were grown at 37 °C in a 5 % CO_2_ atmosphere in Dulbecco's modified Eagle's medium (DMEM) supplemented with 10 % heat-inactivated fetal bovine serum (FBS; Gibco), 100 U penicillin ml^−1^ (Gibco) and 100 μg streptomycin ml^−1^ (Gibco). The source of VACV strain Western Reserve (WR) vΔB8R and CMLV strain CMS was described previously (Gubser & Smith, 2002; Symons *et al.*, 2002).

### Viral growth curves.

The one-step growth kinetics of VACV strains were determined as described previously (Pires de Miranda *et al.*, 2003).

### Plasmids.

CMLV strain CMS genomic DNA was used for PCR-mediated amplification of the *v-slfn* gene. For construction of recombinant viruses, haemagglutinin (HA)-tagged or wild-type (WT) v-slfn were cloned into pMJ601 (Davison & Moss, 1990) to create plasmids pMJ601-176RWT, pMJ601-176RNHA and pMJ601-176RCHA. Oligonucleotide primers were forward primers 176FW (5′-CCCCCGCTCGAGGCCGCCACCATGGCGATGTTTTACGCACACGC-3′) and 176FH (5′-CCCCCGCTCGAGGCCGCCACCATGTACCCATACGATGTTCCAGATTACGCTGCGATGTTTTACGCACACGC-3′), and reverse primers 176RW (5′-CGCCGCCCCGGGTTAAAATTTTATAGATGACACCC-3′) and 176RH (5′-CGCCGCCCCGGGTTAAGCGTAATCTGGAACATCGTATGGGTAAAATTTTATAGATGACACCC-3′); restriction sites for *Xho*I and *Sma*I, respectively, are underlined. The influenza virus A/34/PR/8 HA gene was subcloned from plasmid pGS63 (Smith *et al.*, 1987) into pMJ601 to create plasmid pMJ601-H1. For bacterial expression, the *v-slfn* gene was cloned into the pET28a vector (Novagen) without the stop codon to obtain a C-terminal histidine (His) tag, creating pET28-176CHis. Oligonucleotide primers were 176FB (5′-CCCCCCCATGGCGATGTTTTACGCACACGC-3′) and 176RB (5′-CCCCCCTCGAGAAATTTTATAGATGACACCC-3′); restriction sites for *Nco*I and *Xho*I, respectively, are underlined. For production of stable cell lines, the *v-slfn* gene encoding a C-terminal FLAG tag was cloned into the T-REx tetracycline-inducible expression vector pcDNA4/TO (Invitrogen) creating pcDNA4/TO-v-slfn-FLAG. Oligonucleotide primers were CMLV176 forward (5′-CGCTCTAGAATGGCGATGTTTTACGCA-3′) and CMLV176 reverse (5-CCCAAGCTTTTA**CTTGTCGTCGTCGTCCTTGTAGTC**AAATTTTATAGATGACAC-3′). Underlined and bold nucleotides represent the restriction site for *Hin*dIII and FLAG tag, respectively. The fidelity of all PCR-generated DNA sequences was confirmed by sequencing.

### Generation of v-slfn tetracycline-inducible stable cells.

NIH3T3 murine fibroblasts were transfected with linearized pcDNA4/TO-v-slfn-FLAG and pcDNA6/TR vectors (Invitrogen) and cell lines were selected under antibiotic selection with 3 μg blasticidin ml^−1^ and 750 μg zeocin ml^−1^ for 3 weeks. Clone B7 was selected as the line exhibiting no basal v-slfn expression and the highest tetracycline inducibility.

### Production of rabbit polyclonal antiserum to v-slfn.

Plasmid pET28-176CHis was transformed into Rosetta-gami *Escherichia coli* cells (Novagen) and cultured in Luria–Bertani (LB) medium at 37 °C until the OD_600_ reached 0.6. Protein expression was induced by the addition of 1 mM IPTG for 3 h at 37 °C. Cells were lysed in BugBuster protein extraction reagent (Novagen) containing protease inhibitor cocktail set III (Calbiochem). The bacterial lysates were sonicated and the insoluble material was removed by centrifugation at 10 000 ***g*** for 10 min. v-slfn was purified from insoluble inclusion bodies by washing four times in wash buffer (20 mM Tris pH 7.5, 10 mM EDTA, 1 % Triton X-100). SDS-PAGE and Coomassie blue staining confirmed that v-slfn was the major protein present (data not shown). A polyclonal rabbit antiserum was raised against v-slfn by Harlan Seralabs. To reduce cross-reactivity of the antiserum with denatured mammalian proteins, the antiserum was incubated with HeLa cell proteins. HeLa cells (3×10^8^) were lysed using acetone and proteins were collected by centrifugation and dried overnight at room temperature. Antiserum diluted 1 : 10 in PBS was added to the precipitate and incubated with shaking for 48 h. The protein precipitate was removed by centrifugation and filtration, and the supernatant was used for immunoblotting.

### Immunoblotting.

BS-C-1 cells were mock-infected or infected at 5 p.f.u. per cell for the indicated time and lysed in radioimmunoprecipitation assay (RIPA) buffer. Protein samples were resolved by SDS-PAGE, transferred to a nitrocellulose membrane and probed with *α*-v-slfn polyclonal rabbit antiserum (1 : 100), rat monoclonal antibody (mAb) 15B6 against the VACV F13 protein (1 : 100; Schmelz *et al.*, 1994), mouse *α*-FLAG M2 mAb (1 : 1000; Sigma-Aldrich) or mouse *α*-HA mAb (1 : 1000; Covance). Secondary antibodies were horseradish peroxidase (HRP)-conjugated goat anti-rabbit, anti-rat and anti-mouse IgG (1 : 2000; Sigma-Aldrich). Bound Ab was detected using Enhanced Chemiluminescence Plus Western blotting detection reagents (Amersham Biosciences).

### Immunofluorescence.

HeLa cells were grown on sterile glass coverslips (borosilicate glass; BDH) in six-well plates and intracellular proteins were stained as described previously (Law *et al.*, 2004). Samples were examined with a Zeiss LSM 510 laser scanning confocal microscope. Images were captured and processed using Zeiss LSM Image Browser version 3.2. Primary Abs used were *α*-v-slfn polyclonal antiserum (1 : 200) and *α*-HA mAb (1 : 500).

### Construction of recombinant viruses.

VACV vΔB8R was used as the parental virus for construction of recombinant VACV expressing WT v-slfn, HA-tagged v-slfn or influenza virus HA from the TK locus. CV-1 cells were infected with vΔB8R at 0.1 p.f.u. per cell for 1 h and then transfected with pMJ601, pMJ601-176RWT, pMJ601-176RNHA, pMJ601-176RCHA or pMJ601-H1. Virus was harvested from infected cells 3 days later and recombinant TK^−^ viruses were selected by growth on TK^−^143 cells in the presence of 25 μg 5-bromodeoxyuridine (BUdR) ml^−1^ and chromogenic substrate X-Gal, as described previously (Chakrabarti *et al.*, 1985). Blue plaques were picked, plaque purified and the presence of v-slfn was confirmed by PCR and immunoblotting. Resulting viruses were named vΔB8R, v176-WT, v176-NHA, v176-CHA and vH1, respectively.

### Mouse intradermal and intranasal model of infection.

The intradermal inoculations were carried out as described previously (Tscharke & Smith, 1999). For the intranasal model, groups of female BALB/c mice (6–8 weeks old) were infected intranasally with 4×10^6^ or 10^7^ p.f.u. sucrose-purified virus in 20 μl PBS, and their weight and signs of disease were scored daily as described previously (Alcami & Smith, 1992). Mice infected with 4×10^6^ p.f.u. vH1, v176-WT or v176-NHA were sacrificed at 3, 5 and 7 days post-infection (p.i.). Cells present in the alveoli were removed by bronchoalveolar lavage (BAL) using BAL solution (12 mM lidocaine and 5 mM EDTA in Earl's balanced salt solution), centrifuged at 800 ***g***, resuspended in erythrocyte lysis buffer (0.829 % NH_4_Cl, 0.1 % KHCO_3_, 0.0372 % Na_2_EDTA) for 3 min and kept on ice in RPMI/10 % FBS. Lung cells were obtained from lung homogenates by enzymic digestion, lysis of erythrocytes and centrifugation through 20 % Percoll (Sigma-Aldrich), as described previously (Clark *et al.*, 2006). Live cells in BAL and lung single-cell suspensions were counted, blocked and stained with appropriate combinations of fluorescein isothiocyanate-, phycoerythrin- or tricolour-labelled *α*-CD25, *α*-CD69, *α*-CD3, *α*-CD8, *α*-CD4, B220 or *α*-DX5 and the relevant isotype mAb controls (BD Biosciences) as described previously (Clark *et al.*, 2006). The distribution of cell-surface markers was determined on a FACScan flow cytometer with CellQUEST software (BD Biosciences). A lymphocyte gate was used to analyse data from at least 20 000 events. The titres of virus in tissues were determined as described previously by plaque assay on duplicate monolayers of TK^−^143 cells (Reading & Smith, 2003).

### Statistical analysis.

Student's *t*-test (two-tailed) was used to test the significance of the results.

## RESULTS

### CMLV protein 176 (v-slfn) is related to members of the slfn family

The characterization of murine slfn1 (m-slfn1), the prototype of the slfn protein family, led to the identification of related sequences in several OPVs (Schwarz *et al.*, 1998). Subsequent sequencing of CMLV identified another v-slfn orthologue (protein 176, called v-slfn hereafter) (Gubser & Smith, 2002). CMLV v-slfn was predicted to encode a 502 aa protein with an N-terminal domain that has 22 % amino acid identity (approx. 41 % similarity) to an uncharacterized family of baculovirus proteins, collectively known as p26 (Liu *et al.*, 1986), and a C-terminal domain related to the conserved region of m-slfns (Fig. 1). v-slfn is poorly conserved amongst chordopoxviruses, with only members of the OPV genus encoding putative v-slfn counterparts; however, a p26-like domain is present in certain entomopoxvirus proteins. *Melanoplus sanguinipes* entomopoxvirus (MSEV) ORF 237 encodes a protein with 43 % identity and 61 % similarity to the first 197 aa of CMLV v-slfn, although this sequence is absent in *Amsacta moorei* entomopoxvirus (AMEV) (Afonso *et al.*, 1999; Bawden *et al.*, 2000; Tulman *et al.*, 2006).

Other OPVs predicted to encode full-length v-slfns are CPXV, ectromelia virus (ECTV), monkeypox virus (MPXV) and taterapox virus (GBLV) (http://www.poxvirus.org), whereas in VACV and VARV, the corresponding gene is broken into fragments. All full-length OPV v-slfns share between 84 and 100 % amino acid identity (93–100 % amino acid similarity) with CMLV strain CMS v-slfn. Fig. 1(a) shows a depiction of CMLV v-slfn protein and its relationship with the v-slfn fragments from VACV strain Western Reserve (WR) and a representative member of each of the m-slfn subgroups. Altogether, CMLV v-slfn is most similar to mammalian short m-slfns (m-slfn1 and 2), lacking the C-terminal extensions of intermediate (m-slfn3 and 4) and long (m-slfn5, 8, 9, 10 and 14) m-slfns. Fig. 1(b) shows an alignment of the conserved region of m-slfns with the C terminus of v-slfn. In this region, v-slfn shares between 24 and 30 % identity (42–60 % similarity) with m-slfns. Notably, v-slfn lacks similarity to the first 27 aa of m-slfn1, which are essential for m-slfn1-mediated inhibition of cell growth of fibroblasts (Fig. 1b[Fig f1]; Geserick *et al.*, 2004). The unrooted tree in Fig. 1(c) shows the phylogenetic relationships of OPV v-slfn proteins with their mammalian counterparts. Due to their high sequence similarity, all OPV v-slfns group closely together. Compared to mammalian counterparts, v-slfns are most closely related to short m-slfns (m-slfn1 and 2) and least related to long m-slfns (m-slfn5, 8, 9, 10 and 14), due to their lack of C-terminal extension and also because of a lower degree of similarity within the m-slfn conserved region (Fig. 1b[Fig f1]). Fig. 1(c) also highlights the reported divergence of m-slfn5 protein sequence (Geserick *et al.*, 2004) and that of the newly identified m-slfn14 (GenBank accession no. XP_899217) to other long m-slfns.

### Expression of v-slfn in fibroblasts

**Fig. 2. f2:**
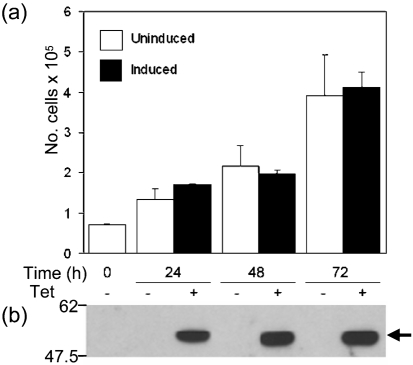
v-slfn effect on NIH3T3 cell growth. (a) NIH3T3 cell line (B7) expressing v-slfn stably upon tetracycline induction was seeded at a density of 5×10^5^ cells per 100 mm dish in triplicate and, after 24 h, expression of v-slfn was induced or not by addition of tetracycline (0.5 μg ml^−1^). Cells were cultured over 72 h, harvested by trypsinization and counted in duplicate. (b) Cell lysates from (a) were analysed for expression of v-slfn using an *α*-FLAG mAb (arrow). Data are representative of three independent experiments. Positions of molecular size markers (kDa) are indicated.

v-slfn is most similar to short m-slfns, of which m-slfn1 inhibits fibroblast cell growth *in vitro* (Schwarz *et al.*, 1998; Brady *et al.*, 2005). To investigate whether v-slfn had a similar effect on cell growth, a C-terminally FLAG-tagged v-slfn was expressed stably in NIH3T3 cells using the T-REx inducible system (Methods). Two independent clones (clone B7; Fig. 2 and data not shown) were induced for expression of v-slfn and after 24, 48 and 72 h the number of viable cells was assessed (Fig. 2a[Fig f2]). Although v-slfn was expressed after addition of tetracycline (Fig. 2b[Fig f2], arrow), there was no difference in cell proliferation between cells that did or did not express v-slfn, showing that unlike m-slfn1, but like intermediate long m-slfns (Schwarz *et al.*, 1998; Geserick *et al.*, 2004; Brady *et al.*, 2005), v-slfn does not affect fibroblast cell growth *in vitro.*

### Detection and characterization of v-slfn

**Fig. 3. f3:**
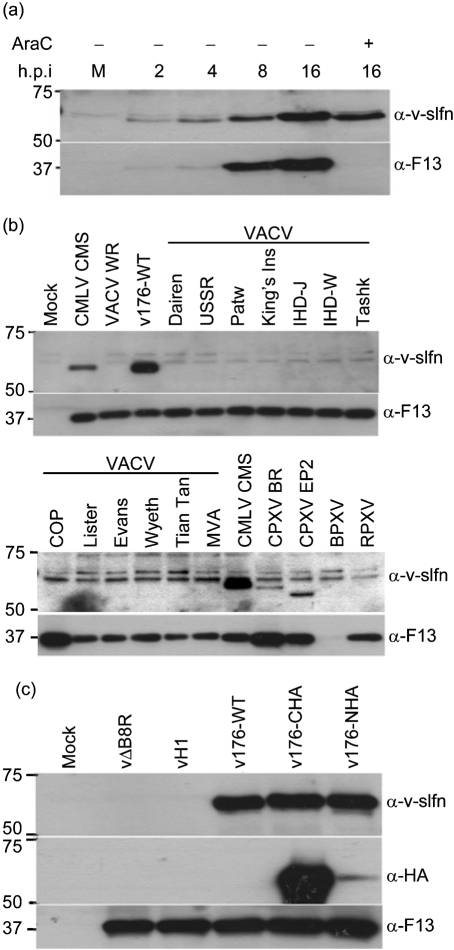
Characterization of v-slfn by immunoblotting. (a) HeLa cells were either mock-infected (M) or infected with CMLV strain CMS at 5 p.f.u per cell and harvested when indicated. + indicates that AraC was present throughout infection. Cell extracts were analysed by immunoblotting using either *α*-v-slfn or *α*-F13 Abs. (b) Expression of v-slfn protein in OPVs. Monolayers of BS-C-1 cells were mock-infected or infected with 5 p.f.u. of the indicated virus per cell, harvested at 6 h p.i. and cell extracts were analysed by immunoblotting using either *α*-v-slfn or *α*-F13 Abs. CMLV, camelpox virus; VACV, vaccinia virus; WR, Western Reserve; Patw, Patwadangar; King's Ins, King's Institute; Tashk, Tashkent; COP, Copenhagen; CPXV, cowpox virus; EP2, elephantpox virus-2; BPXV, buffalopox virus; RPXV, rabbitpox virus. (c) HeLa cells were either mock-infected or infected with 10 p.f.u. of the indicated VACV per cell, harvested 16 h p.i. and cell extracts were analysed by immunoblotting. In all panels, the positions of molecular size markers (kDa) are indicated.

To characterize v-slfn, the CMLV *176R* gene was expressed in *E. coli* with a C-terminal His tag, and the recombinant protein was purified from inclusion bodies and used to raise a rabbit *α*-v-slfn polyclonal Ab (Methods). In immunoblots, the *α*-v-slfn Ab detected an approximately 57 kDa protein in cells infected with CMLV (Fig. 3a, b[Fig f3]) or with a recombinant VACV expressing CMLV v-slfn with or without an HA tag (Fig. 3b, c[Fig f3]). v-slfn was not present in mock-infected cells.

The time of expression of the v-slfn protein during infection was investigated in CMLV-infected cells (Fig. 3a[Fig f3]). v-slfn was expressed from 2 h p.i. and its expression levels increased until 16 h p.i. The protein was also detected at 16 h p.i. in cells in the presence of cytosine arabinoside (AraC), an inhibitor of DNA replication and thereby of virus intermediate and late gene expression, indicating that v-slfn was expressed early during infection. The higher level of expression of v-slfn in the absence of AraC indicates that the protein continues to be made after DNA replication has started. In contrast, a 37 kDa protein, which is the orthologue of VACV late protein F13, was not expressed in the presence of AraC (Fig. 3a[Fig f3]).

Sequence data predict that a v-slfn is expressed by some OPVs; however, the v-slfn ORF is broken in sequenced VACV and VARV strains (http://www.poxvirus.org). To analyse whether other VACV and CPXV strains express a full-length v-slfn, BS-C-1 cells were infected with 16 strains of VACV and two strains of CPXV, and cell extracts were analysed by immunoblotting (Fig. 3b[Fig f3]). This showed that none of the VACV strains examined expressed a protein detected by the *α*-v-slfn Ab. However, in accord with available sequence data, v-slfn was detected in cells infected with CPXV strain Brighton Red (BR). Similarly, elephantpox virus (EP2), another CPXV strain, also expressed a v-slfn counterpart. For both CPXV strains, the proteins migrated slightly faster than CMLV v-slfn, despite having a comparable predicted molecular mass (CPXV strain BR; http://www.poxvirus.org). Both CPXV v-slfns were detected at lower levels (Fig. 3b[Fig f3]); however, this might be due to *α*-v-slfn Ab specificity. The recognition of a 37 kDa protein (VACV F13 orthologue) by an F13-specific mAb confirmed that all cells had been infected (Fig. 3b[Fig f3], lower panels). Note that the F13 orthologue in buffalopox virus (BPXV)-infected cells was only detected at later times p.i. (data not shown) and that a non-specific doublet is present in mock-infected and infected cells. Thus, v-slfn is an early and late protein that is encoded by a full-length ORF in several OPVs, but is not expressed by any of the 16 VACV strains tested, suggesting that it is either absent or broken into smaller ORFs, as is the case for VACV strains Copenhagen (COP) and WR.

### Construction of recombinant VACV expressing CMLV v-slfn

A VACV strain expressing full-length v-slfn was not identified, therefore the function of v-slfn was studied using a recombinant VACV expressing the CMLV v-slfn. An attenuated VACV strain lacking the *B8R* gene encoding the gamma interferon-binding protein (vΔB8R) (Symons *et al.*, 2002) was selected as the parent virus to meet biosafety concerns that insertion of the CMLV *176R* gene into VACV might increase virulence. In addition, the CMLV *176R* gene was inserted into the TK locus of vΔB8R to provide a second attenuating mutation (Buller *et al.*, 1985). v-slfn was expressed either without (v176-WT) or with an N- or C-terminal HA tag (v176-NHA and v176-CHA, respectively) under the control of a synthetic early and late promoter. v-slfn expression by these viruses was observed from 2 h p.i., similar to the expression pattern seen in CMLV-infected cells (Fig. 3a[Fig f3] and data not shown). A control virus expressing the HA from influenza A/PR/8/34 (Bennink *et al.*, 1986) from the same locus and using the same promoter was also constructed (vH1). The genotype of recombinant viruses was confirmed by PCR (data not shown). HeLa cells were mock-infected or infected with vΔB8R, vH1, v176-WT, v176-NHA or v176-CHA, and expression of v-slfn was detected using *α*-v-slfn Ab. Fig. 3(c) shows that v176-WT, v176-CHA and v176-NHA express v-slfn whereas, vΔB8R and vH1 do not (top panel). Infection was confirmed by detection of the VACV F13 orthologue in all samples in parallel blots (Fig. 3c[Fig f3], bottom panel). The blots were then stripped and reprobed with an *α*-HA mAb (Fig. 3c[Fig f3], middle panel). This detected the HA-tagged protein expressed by v176-CHA and 176-NHA. There was, however, a reproducible large difference in level of detection between the two samples, suggesting that the N terminus of N-terminally tagged v-slfn is removed by proteolytic cleavage.

To determine whether expression of v-slfn altered virus replication, the growth properties of vΔB8R, vH1, v176-WT, v176-NHA and v176-CHA were analysed. After infection of BS-C-1 cells at 10 p.f.u. per cell for 24 or 48 h, the total virus yield of all recombinant viruses was indistinguishable (Supplementary Fig. S1, available in JGV Online). Similarly, VACV plaque morphology and size were unaltered by expression of v-slfn in BS-C-1 (Supplementary Fig. S2), RK_13_ and TK^−^143 cells (data not shown). Therefore, v-slfn does not affect virus growth or plaque size when expressed by recombinant VACV under the conditions tested.

### Subcellular localization of v-slfn

**Fig. 4. f4:**
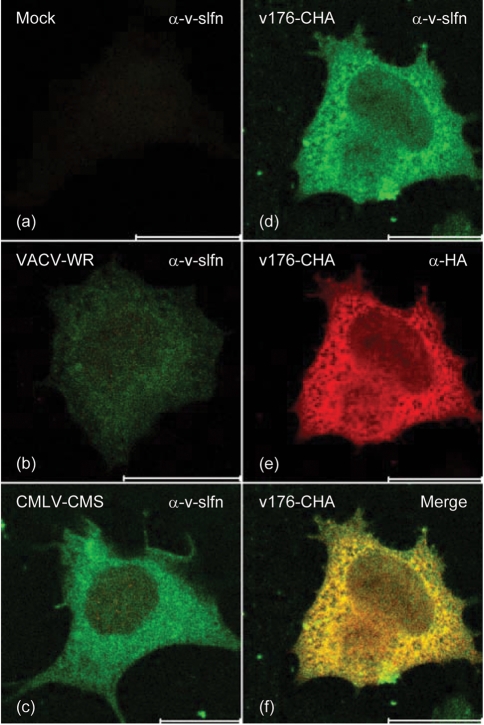
Subcellular localization of v-slfn. HeLa cells were either (a) mock-infected or infected with 5 p.f.u. per cell of (b) VACV WR, (c) CMLV-CMS or (d–f) v176-CHA for 6 h and were analysed by immunofluorescence using *α*-v-slfn (a–d) or *α*-HA (e) Abs. (f) Shows a merged image of (d) and (e). Bars, 20 μm.

To investigate the subcellular localization of v-slfn, HeLa cells were either mock-infected or infected with CMLV strain CMS, VACV WR or v176-CHA for 6 h (Fig. 4). v-slfn was detected as a predominantly cytoplasmic protein with no obvious localization to any organelles (Fig. 4c–e[Fig f4]) and similar localization was detected at later time points (data not shown). No signal was present in mock-infected cells (Fig. 4a[Fig f4]), and only a weak background was present in cells infected with VACV WR (Fig. 4b[Fig f4]). The specificity of the v-slfn antiserum was confirmed by co-staining cells infected with v176-CHA with *α*-v-slfn Ab (Fig. 4d[Fig f4]) and *α*-HA mAb (Fig. 4e[Fig f4]) and merging the images (Fig. 4f[Fig f4]), showing co-staining with these two Abs. v-slfn localization was also analysed in NIH3T3 cell lines expressing v-slfn using an *α*-FLAG mAb (data not shown), and after transient transfection of HeLa cells with the mammalian expression vector pCI alone or pCI expressing v-slfn with or without an HA tag. v-slfn was visualized with either *α*-v-slfn antiserum or *α*-HA mAb. All approaches showed the same predominantly cytoplasmic localization for v-slfn, ruling out the possibility that other factors associated with virus infection contributed to v-slfn localization (data not shown).

### Expression of v-slfn attenuates VACV in the murine intranasal model

**Fig. 5. f5:**
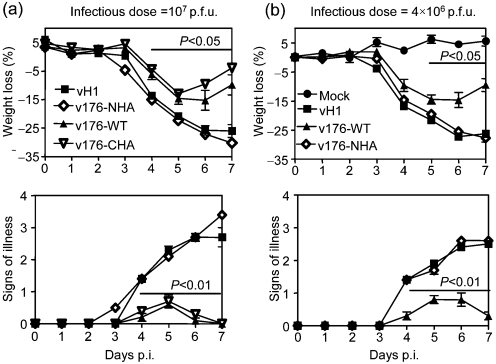
Virulence assay using the intranasal model of infection. Groups of five BALB/c mice were mock-infected or infected with (a) 10^7^ or (b) 4×10^6^ p.f.u. of the indicated purified viruses. Mice were weighed daily, and results are the mean percentage weight change of each group ± sem compared to the weight on the day of infection (upper panels). Animals were monitored daily for signs of illness, scored 1 to 4 (Alcami & Smith, 1992) (lower panels). Data are expressed as the mean ± sem. *P* values were determined using the Student's *t*-test and indicate the mean percentage weight changes or signs of illness that were significantly different between groups.

The virulence of recombinant VACV that does or does not express v-slfn was examined in the murine intradermal and intranasal models of infection. In the intradermal model, no difference in lesion size was observed between recombinant VACV expressing v-slfn or not (Supplementary Fig. S3, available in JGV Online). In the murine intranasal model of infection, mice infected with 10^7^ p.f.u. vH1, v176-WT, v176-NHA or v176-CHA all showed loss of body weight (Fig. 5a[Fig f5], upper panel) and signs of illness (Fig. 5a[Fig f5] lower panel); however, from day 4 p.i. there was a significant attenuation (*P*<0.05) of v176-WT and v176-CHA compared with vH1 and v176-NHA. In addition, mice infected with v176-WT or v176-CHA started to recover sooner after infection (day 6, Fig. 5a[Fig f5]) compared with mice infected with vH1 and v176-NHA. This showed that (i) expression of v-slfn attenuated VACV in this model, and (ii) the function of v-slfn was affected by the HA tag at the N terminus. The expression of the influenza virus H1 HA from the TK locus did not alter VACV virulence in this model (data not shown).

**Fig. 6. f6:**
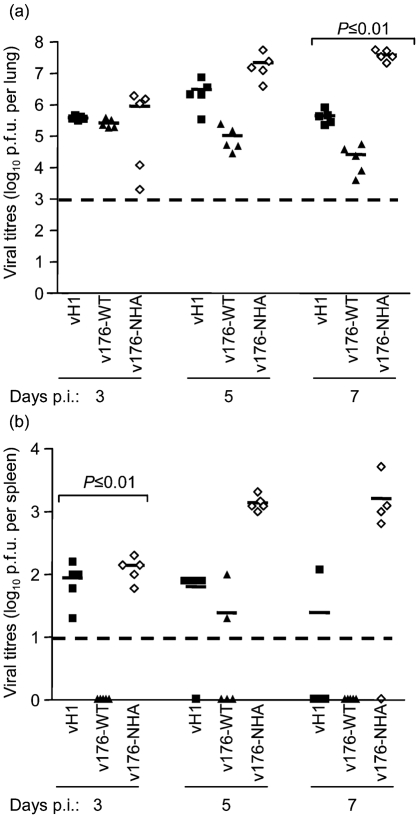
Virus titres of vH1, v176-WT and v176-NHA in (a) lungs and (b) spleens. Groups of five mice were infected intranasally with 4×10^6^ p.f.u. of the indicated virus, and the lungs and spleen were harvested at days 3, 5 and 7 p.i. Virus titres were determined by plaque assay on TK^−^143 cells. Virus titres are expressed as the mean log_10_ p.f.u. per organ ± sem. *P* values were determined using Student's *t*-test and indicate the mean virus titres that were significantly different between mice infected with v176-WT and mice infected with v176-NHA and vH1. Broken line indicates the minimum detection limit of the plaque assay.

To study v-slfn-mediated virus attenuation, recombinant VACV expressing an irrelevant protein (vH1) or non-attenuating v-slfn (v-176NHA) were used as controls. Mice were infected with 4×10^6^ p.f.u. vH1, v176-WT or v176-NHA (Fig. 5b[Fig f5]) and virus titres were measured in lungs (Fig. 6a[Fig f6]) and spleens (Fig. 6b[Fig f6]) of infected animals. Initially, all viruses replicated similarly in the lungs (day 3), but from day 5 there was a reduction in virus titre after infection with v176-WT, compared with v176-NHA and vH1. This difference was significant at day 7 p.i. for both control groups (Fig. 6a[Fig f6]). In the spleen, the v176R-WT titre was below the level of detection at days 3 and 7 p.i., unlike vH1 and v176-NHA, indicating that v176R-WT spread to this organ later and was cleared earlier than control viruses (Fig. 6b[Fig f6]).

**Fig. 7. f7:**
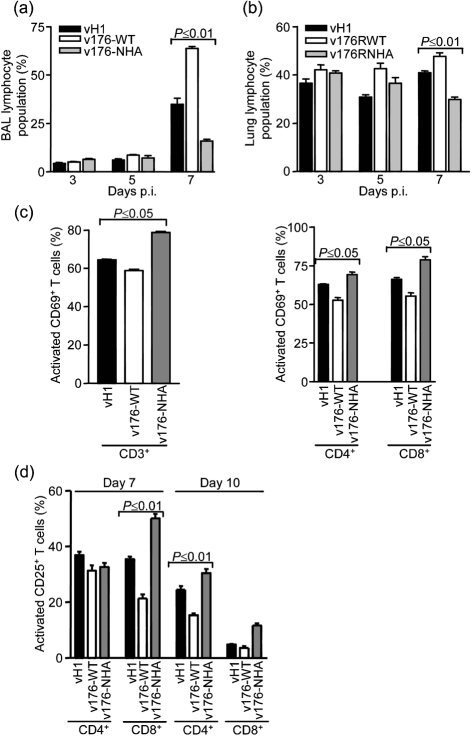
Analysis of cellular inflammatory response by flow cytometry. (a, c and d) BAL and (b) lung cells were recovered on the indicated day (a, b and d) or at day 7 (c) p.i. (a, b) Lymphoid population was determined by its characteristic forward and side scatter profiles. (c, d) Percentage of cells expressing activation markers CD69^+^ or CD25^+^ in CD3^+^, CD4^+^ and CD8^+^ cell populations. Data shown are the mean percentage from five or six mice ± sem. *P* values were determined using Student's *t*-test and indicate the mean data that were significantly different between mice infected with v176-WT, mice infected with v176-NHA and mice infected with vH1. To obtain sufficient cells in BALs, cells from two mice were pooled.

The early clearance of virus after infection with v176-WT compared with control groups suggested a more effective antiviral host response, and therefore the cellular inflammatory response in lungs was analysed by flow cytometry (Fig. 7). At days 3, 5 and 7 p.i., cells recovered from BALs and lung cells from mice infected with v176-WT contained an increased number of lymphoid cells compared with mice infected with control viruses, and this difference was significant at day 7 p.i. in BALs (Fig. 7a[Fig f7]) and lungs (Fig. 7b[Fig f7]). To test whether this increase in lymphoid cells was due to an enhanced recruitment of a particular lymphoid subset, the percentage of CD4^+^ and CD8^+^ T lymphocytes, B220^+^ B lymphocytes and DX5^+^ natural killer (NK) cells present in lungs was analysed, but no significant difference between groups of mice was found (data not shown). Next, the activation of CD3^+^ T cells was investigated using the activation marker CD69^+^. This showed that although on day 7 p.i. there were more lymphocytes recruited to lungs infected with v-176WT compared with infection with vH1 or v176-NHA, CD3^+^ T cells were less activated (Fig. 7c[Fig f7], left panel), and this was reflected by a lower level of the activation marker CD69^+^ on both CD4^+^ and CD8^+^ lymphocytes (Fig. 7c[Fig f7], right panel). There was no difference between groups in CD3^+^ CD69^+^ lymphocytes at days 3 and 5 p.i. (data not shown). Another activation marker, CD25^+^, was used in a further experiment and showed that at days 7 and 10 p.i. there were also less CD25^+^ CD4^+^ and CD25^+^ CD8^+^ lymphocytes in BALs of mice infected with v-176WT compared with control groups, although this difference was only significant for CD8^+^ lymphocytes at day 7 and CD4^+^ lymphocytes at day 10 (Fig. 7d[Fig f7]).

## DISCUSSION

This paper provides a characterization of the CMLV-CMS *176R* gene, which is shown to encode a predominantly cytoplasmic 57 kDa protein that is expressed both early and late in infection. v-slfn shows sequence similarity to the mammalian schlafen protein family and is most closely related to the short members, m-slfn1 and 2. Construction of an inducible cell line that expresses v-slfn showed that, unlike m-slfn1, v-slfn did not inhibit NIH3T3 cell growth. This is probably due to v-slfn lacking a region with sequence similarity to m-slfn1 aa 1–27 that is essential for m-slfn1-mediated inhibition of fibroblast cell growth (Geserick *et al.*, 2004).

The m-slfn proteins are expressed differentially in activated T cells and macrophages, and regulate T-cell development and differentiation (Schwarz *et al.*, 1998; Geserick *et al.*, 2004). Since there is no established small animal model for studying CMLV pathogenesis, we screened several VACV strains, hoping to identify a strain that expresses a full-length v-slfn. However, none of the 16 strains examined expressed a v-slfn orthologue suggesting that, like VACV strain COP, the v-slfn ORF is broken into smaller fragment in these viruses. Sequence data from VACV strains WR, MVA and Lister are consistent with these observations. Therefore, the function of v-slfn *in vivo* was investigated using a recombinant VACV expressing untagged v-slfn (v176WT) or v-slfn with a C-terminal (v176-CHA) or N-terminal (v176-NHA) HA tag. Expression of WT or tagged v-slfn by recombinant VACV did not affect virus virulence in the intradermal model of infection. However, expression of WT v-slfn or C-terminally tagged v-slfn attenuated VACV in a murine intranasal model, resulting in reduced weight loss and more rapid recovery compared with control groups. In contrast, N-terminally tagged v-slfn did not attenuate VACV in this model, showing that v-slfn function was affected by addition of an HA tag on the N terminus, and suggesting that this part of the protein is important for function. The altered function of N-terminally tagged v-slfn is possibly due to cleavage of a small part of the N terminus of the protein after addition of the HA tag, as shown by immunoblotting (Fig. 3c[Fig f3]). v-slfn-mediated attenuation *in vivo* was characterized by a significantly reduced virus titre in lungs at day 7 p.i. compared with vH1 and v-176NHA, although at day 3 the titres were equivalent. This shows that expression of v-slfn did not prevent establishment of infection or virus replication, but probably accelerated virus clearance by the immune system. Consistently, v176-WT spread to the spleen was delayed and v176-WT was cleared more rapidly from this organ compared with control groups.

Characterization of the cellular inflammatory response in the lungs showed that v176-WT infection was characterized by a more pronounced recruitment of lymphocytes that was most striking at day 7 p.i. However, in lung cells, no particular lymphoid subset (CD4^+^ or CD8^+^ T cells, B cells or NK cells) was solely responsible for this increase. The mechanism by which v-slfn promotes such vigorous lymphocyte recruitment is under investigation. At day 7 p.i., when mice infected with v176-WT had almost recovered, but mice infected with vH1 and v176-NHA were still getting sicker, the CD3^+^ lymphocytes present in the BALs of mice infected with v176-WT showed a reduced expression of activation markers CD69 and CD25, compared with control groups. This was consistent with the greater and earlier recruitment of lymphocytes, so that the immune response was already declining by day 7.

It is unclear why a poxvirus would encode a protein that decreases virus virulence. One possibility is to prevent the virus from overwhelming its host too quickly. Another is that the v-slfn protein affects virus virulence differently in other models of infection and, consistent with this, v-slfn did not affect virulence in the intradermal model (Supplementary Fig. S3, available in JGV Online). VACV WR gene *B15R*, which encodes a soluble interleukin-1*β* receptor, is another example of a gene that decreases viral virulence in one model but not others (Alcami & Smith, 1992, 1996; Spriggs *et al.*, 1992; Tscharke *et al.*, 2002; Staib *et al.*, 2005). In summary, we have identified and characterized CMLV strain CMS *176R* and shown that it encodes an intracellular protein, which attenuates VACV *in vivo* but does not affect virus replication or plaque morphology *in vitro*. v-slfn shares sequence similarity with members of the m-slfn family of mammalian proteins, and recent reports suggest that these play a role in the modulation of the innate and adaptive immune responses against pathogens (Schwarz *et al.*, 1998; Eskra *et al.*, 2003; Geserick *et al.*, 2004). The exact role of v-slfn within the context of a viral infection remains to be elucidated.

## Supplementary Material

[Supplementary Figures]
