# Supplementary material for: Camelpox virus encodes a schlafen-like protein that affects orthopoxvirus virulence
Source: J Gen Virol. 2007 Jun;88(Pt 6):1667–76. doi: 10.1099/vir.0.82748-0 (PMC2885618; doi:10.1099/vir.0.82748-0)
Supplement: [Supplementary Figures] [file supp_88_6_1667__index.html]

 Camelpox virus encodes a schlafen-like protein that affects orthopoxvirus virulence -- Gubser et al. 88 (6): 1667 Data Supplement - Supplementary Figures -- Journal of General Virology

## 

### Camelpox virus encodes a schlafen-like protein that affects orthopoxvirus virulence, by Gubser, C., Goodbody, R., Ecker, A., Brady, G., O'Neill, L. A. J., Jacobs, N. and Smith, G. L.

*Journal of General Virology* vol. **88** , part 6, pp. 1667–1676.

**Supplementary Figures S1, S2 and S3.** [Single PDF file] (243 KB)

  
  
