# Supplementary material for: Camelpox virus encodes a schlafen-like protein that affects orthopoxvirus virulence
Source: J Gen Virol. 2007 Jun;88(Pt 6):1667–76. doi: 10.1099/vir.0.82748-0 (PMC2885618; doi:10.1099/vir.0.82748-0)
Supplement: [Supplementary Figures] [file supp_88_6_1667__1.pdf]

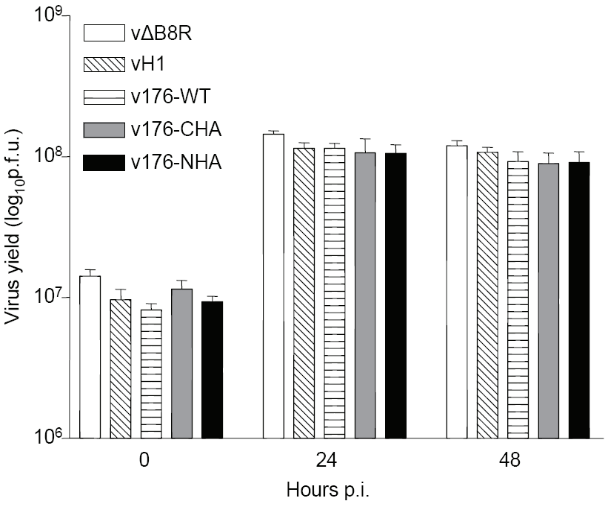

Supplementary Fig. S1. Growth analyses of recombinant VACV in cell culture. BS-C-1 cells were infected with 10 p.f.u. per cell. At indicated time p.i., cells were scraped into the medium and the total infectivity was determined by plaque assay. Data points are the mean titre from triplicate samples ± SEM.

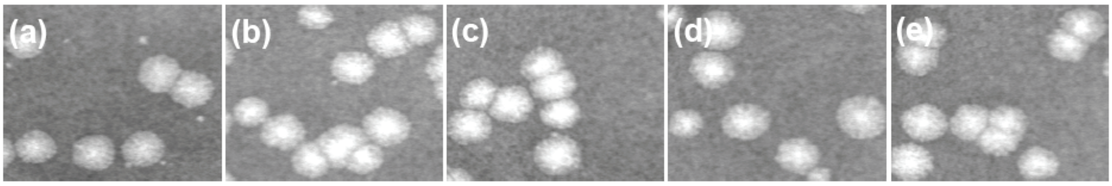

Supplementary Fig. S2. Plaque morphology of recombinant VACV. BS-C-1 cell monolayers were infected with vΔB8R (a), vH1 (b), v176-WT (c), v176-NHA (d) or v176-CHA (e), overlaid with DMEM/2.5 % FBS/1.5 % carboxymethylcellulose and incubated for 3 days at 37 °C. Cells were then stained with 0.1 % (w/v) crystal violet in 15 % (v/v) ethanol.

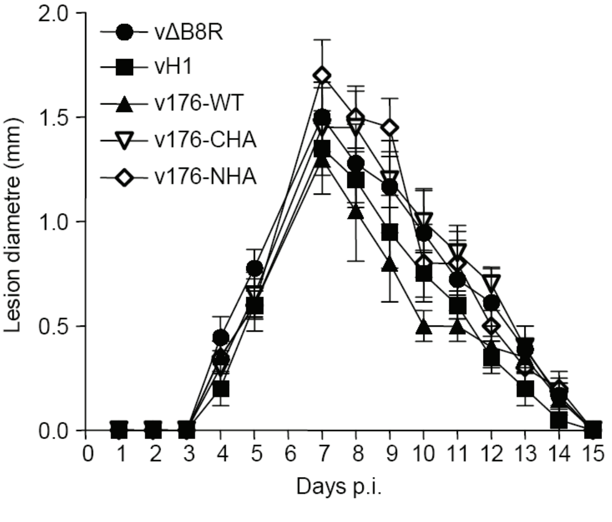

Supplementary Fig. S3. Virulence assay in murine intradermal model. Groups of 5 C57BL/6 mice were infected intradermally in the left ear pinnae with 10^7 p.f.u. of VACV vΔB8R, vH1, v176-WT, v176-NHA or v176-CHA and the lesion diameters were measured daily. Points represent the mean lesion size ± SEM from groups of five mice.

Gubser, C., Goodbody, R., Ecker, A., Brady, G., O'Neill, L. A. J., Jacobs, N. & Smith, G. L. (2007). Camelpox virus encodes a schlafen-like protein that affects orthopoxvirus virulence. *J Gen Virol* **88**, 1667–1676.
